# Emotional disorders in children after acute traumatic injuries

**DOI:** 10.1192/j.eurpsy.2026.10436

**Published:** 2026-07-31

**Authors:** E. Lvova, Y. Sidneva, S. Valiullina

**Affiliations:** 1 neurorehabilitation, Clinical and Research Institute of Emergency Pediatric Surgery and Trauma; 2 neuropsychiatry, N.N.Burdenko National Medical Research Institute of Neurosurgery, Moscow, Russian Federation

## Abstract

**Introduction:**

Acute traumatic injuries in children are varied. These include skeletal trauma, traumatic brain injury, spinal cord injury, amputations, combined trauma, and other injuries. Traumatic injuries often result in severe physical defects, with motor, neurological, and somatic consequences. These defects lead to severe disability and maladjustment in the child. Depressive disorders in children have been described in isolated studies, associated with specific illnesses. It is known that children have difficulty understanding and accepting new living conditions. Physical limitations lead to frustration, feelings of worthlessness, and helplessness; self-esteem and motivation are reduced, complicating rehabilitation measures and the child’s return to their previous environment.

**Objectives:**

To study emotional disorders after traumatic injuries in children during the early stages of rehabilitation.

**Methods:**

165 children under 18 years of age: 80 (severe and moderate TBI), 60 (spinal cord injury), 25 (amputation, skeletal trauma); admitted to the Institute for treatment and rehabilitation. Methods: clinical, psychopathological, psychological. Additionally, data from examinations by neurologists, traumatologists, pediatricians, neuroimaging studies (CT, MRI, ultrasound, and others); scales, questionnaires were used.

**Results:**

Emotional disorders were identified in children after acute injuries: with severe and moderate TBI - in 43% of cases as a consequence of the injury within the framework of psychoorganic syndrome; with spinal cord injury - in 48% as a reaction to a psychotraumatic situation; with amputation, severe skeletal trauma - in 60% as a reaction to a psychotraumatic situation.

During the acute period of traumatic injury, the children underwent comprehensive rehabilitation using a multidisciplinary approach. A neuropsychiatrist recommended neuropharmacotherapy with an antidepressant from the serotonin reuptake inhibitor group (sertraline) and GABAergic drugs. Psychological support was provided using Gestalt correction methods.

**Conclusions:**

Emotional disorders in children are a significant consequence of acute traumatic injuries. The genesis of mental disorders can be caused by both organic damage to brain structures and dysregulation of the nervous system, as well as a reaction to the traumatic situation (the circumstances of the injury, the severity of the illness, and the consequences). To increase the effectiveness of rehabilitation measures, return the child to a normal environment, and improve their quality of life, an interdisciplinary approach to the management of such patients is necessary from the early stages of recovery and rehabilitation.

**Disclosure of Interest:**

None Declared

